# The Processing of Somatosensory Information Shifts from an Early Parallel into a Serial Processing Mode: A Combined fMRI/MEG Study

**DOI:** 10.3389/fnsys.2016.00103

**Published:** 2016-12-20

**Authors:** Carsten M. Klingner, Stefan Brodoehl, Ralph Huonker, Otto W. Witte

**Affiliations:** ^1^Hans Berger Department of Neurology, Jena University Hospital—Friedrich Schiller University JenaJena, Germany; ^2^Biomagnetic Center, Jena University Hospital—Friedrich Schiller University JenaJena, Germany

**Keywords:** MEG, DCM, somatosensory cortex, effective connectivity, perception

## Abstract

The question regarding whether somatosensory inputs are processed in parallel or in series has not been clearly answered. Several studies that have applied dynamic causal modeling (DCM) to fMRI data have arrived at seemingly divergent conclusions. However, these divergent results could be explained by the hypothesis that the processing route of somatosensory information changes with time. Specifically, we suggest that somatosensory stimuli are processed in parallel only during the early stage, whereas the processing is later dominated by serial processing. This hypothesis was revisited in the present study based on fMRI analyses of tactile stimuli and the application of DCM to magnetoencephalographic (MEG) data collected during sustained (260 ms) tactile stimulation. Bayesian model comparisons were used to infer the processing stream. We demonstrated that the favored processing stream changes over time. We found that the neural activity elicited in the first 100 ms following somatosensory stimuli is best explained by models that support a parallel processing route, whereas a serial processing route is subsequently favored. These results suggest that the secondary somatosensory area (SII) receives information regarding a new stimulus in parallel with the primary somatosensory area (SI), whereas later processing in the SII is dominated by the preprocessed input from the SI.

## Introduction

Understanding the process of somatosensory perception requires detailed knowledge of not only the functions of the involved cerebral areas, but also their interactions and particularly the route by which sensory information is transmitted. Although research over the past two decades has significantly improved our understanding of the brain areas involved and their functions, where somatosensory inputs enter the cortical brain matrix and whether the data are processed in a parallel or serial manner remain poorly understood.

The parallel pathway theory proposes that somatosensory inputs project from the thalamus directly to both the primary somatosensory cortex (SI) and the secondary somatosensory cortex (SII) and that these information streams are processed in parallel. The serial pathway theory assumes that there is no direct input of thalamic information to the SII but that somatosensory inputs project from the thalamus to the SI before being relayed to the SII (Rowe et al., 1996). Both theories are supported by anatomical studies that have demonstrated that the SI is connected to the SII via reciprocal cortico-cortical connections (Jones and Powell, 1969) and that different thalamic nuclei project in parallel to the SI and the SII (Almeida et al., 2004). Therefore, pain and tactile information can be conveyed to the SII via an indirect serial pathway from the thalamus via the SI, but this information might also be directly conveyed by the thalamus. However, regardless of whether the anatomical connections allow unaltered input signals to directly or indirectly enter the SII, whether such connections are used to transmit input information or are instead used for top-down modulation remains unknown.

Dynamic causal modeling (DCM) is a method that allows for estimations and inferences about network dynamics to be made based on a Bayesian framework (Friston et al., 2003). Particularly, this method allows for the coupling of a small number of brain areas to be estimated (Friston et al., 2003).

There are four studies available that have applied DCM to fMRI data of somatosensory information processing. However, these studies reached different conclusions regarding whether somatosensory information is processed in parallel or in series. Two of these studies reported evidence supporting parallel processing of somatosensory information (Liang et al., 2011; Chung et al., 2014), while the other two studies reported evidence supporting serial processing of somatosensory information (Kalberlah et al., 2013; Khoshnejad et al., 2014). A recent study that applied DCM analysis to magnetoencephalographic (MEG) data suggested that somatosensory data are processed in parallel during the early stage (within the first 100 ms; Klingner et al., 2015).

**Figure 1:**
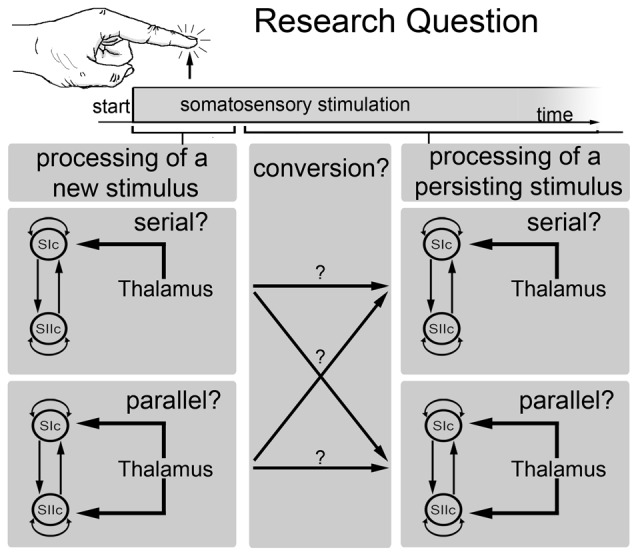
**Schematic outline of the main research question of the current study.** This study investigated whether somatosensory information is processed in parallel or in series and whether the processing mode changes over time. Specifically, whether the processing mode at the beginning of a new stimulus is different from the processing mode of a persisting stimulus.

However, these divergent results could be explained by the hypothesis that the processing route for somatosensory information changes over time. Specifically, we have previously suggested that somatosensory stimuli are processed in parallel only during the early stage and that the processing is later dominated by serial processing (Figure 1). This hypothesis was revisited in the present study based on fMRI analyses of tactile stimuli and the application of DCM to MEG data obtained during sustained (260 ms) tactile stimulation. Bayesian model comparisons were used to make direct inferences regarding the processing stream.

## Materials and Methods

### Subjects

Seventeen healthy volunteers without any histories of neurological or psychiatric diseases participated in this study (mean age 22.9 ± 1.8 years, 9 female). All subjects were right-handed according to the Edinburgh Handedness Inventory (Oldfield, 1971). All subjects provided their written informed consent. This study was approved by the local ethics committee (Ethik-Kommission der Friedrich-Schiller-Universität Jena an der Medizinischen Fakultät) and was performed in accordance with the Human Subjects Guidelines of the Declaration of Helsinki.

### Experimental Protocol

**Figure 2:**
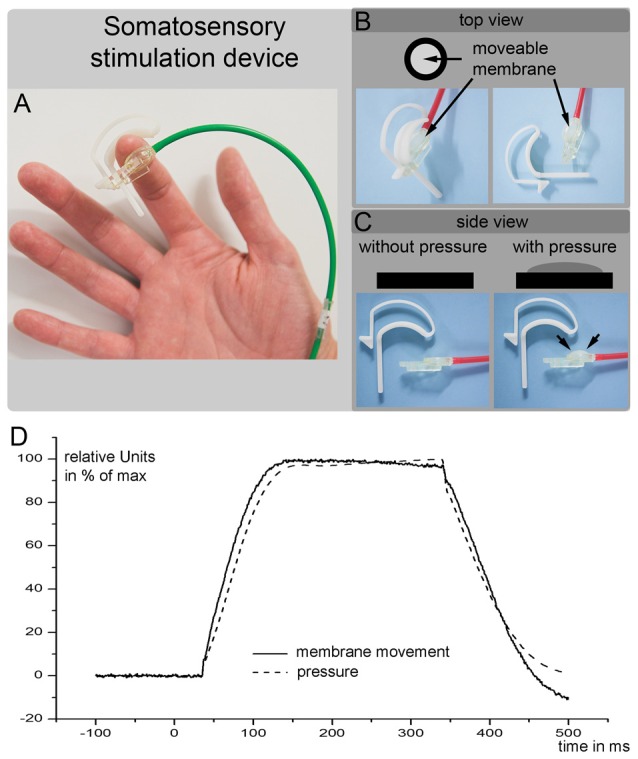
**(A–C)** are a schematic outline of the somatosensory stimulation device used in the current study. **(D)** Shows the movement of the balloon diaphragm (solid line) and the changes in pressure (dotted line). The air pump was started at time 0 and required 40 ms increase the air pressure at the membrane. The *y*-axis shows the changes in the membrane position and changes in the pressure in relative units (maximal movement or pressure = 100%).

All subjects underwent a tactile stimulation paradigm during fMRI image acquisition and during MEG scans. The tactile stimuli were delivered to fingers 1 + 3 of the right hand by balloon diaphragms that were driven by compressed air. A schematic of the device is shown in Figures 2A,B. The movement of the balloon diaphragms and the changes in pressure are illustrated in Figure 2C. The detection threshold of the tactile stimuli was approximately 10% of the stimulation strength used. Therefore, we used a well perceivable but non-painful tactile stimulus. The timings and the durations of the stimuli differed between the fMRI and MEG measurements.

### fMRI Somatosensory Stimulation Procedures

A total of 60 tactile stimuli were presented in an event-related regime (2 s on). Each tactile stimulus consisted of 10 sub-stimuli that were applied by the balloon diaphragm. The event-related interstimulus time was randomized between 7.7 s and 12.8 s. The timings of the stimulus presentations were externally controlled by the MR scanner and were synchronized with the image acquisition.

### MEG Somatosensory Stimulation Procedures

The MEG somatosensory stimulation employed the same tactile stimulation device used during the fMRI experiment. However, the stimulation procedure differed in terms of the timings of the stimuli (Figures 2A–C). During the MEG experiment, 154 tactile stimuli were delivered to fingers 1 + 3 of the right hand in an event-related regimen. Each stimulus lasted for 260 ms. The event-related interstimulus time was randomized between 2 s and 3.8 s. Due to the use of compressed air, there was a time delay between the start of the trigger (i.e., the start of the pump) and the somatosensory stimulation. The diaphragm connected to the skin of the subject’s finger began to rise 35 ms after the pump was triggered (Figure 2D). To ensure a significant increase in the diaphragm, we added 5 ms and considered this time (40 ms) as the onset of the tactile stimulus.

### fMRI Recordings

The images were acquired using a 3.0 tesla MR scanner (Trio, Siemens, Erlangen, Germany). We obtained echo-planar T2*-weighted image volumes (EPI) and transaxial T1-weighted structural images. The functional data were acquired in an EPI session of 230 volumes. The first three EPI volumes were discarded due to equilibration effects. Each functional image volume was comprised of 44 transaxial slices that were obtained during the stimulus paradigm. The scans included the entire cerebrum and cerebellum (voxel size = 3 mm × 3 mm × 3 mm, repetition time = 2.5 s, TE = 35 ms). The high-resolution, T1-weighted structural images had a voxel size of 1 mm × 1 mm × 1 mm to allow for precise anatomical localization.

### fMRI Data Analysis

The data analysis was performed on a workstation using MATLAB (Mathworks, Natick, MA, USA) and SPM12 software (Wellcome Department of Cognitive Neurology, London, UK^1^). For each subject, all images were realigned to the first volume using a six-parameter, rigid-body transformation to correct for motion artifacts. The images were co-registered with the subject’s corresponding anatomical (T1-weighted) images, resliced to correct for acquisition delays, normalized to the Montreal Neurological Institute (MNI) standard brain (Evans et al., 1993) to report the MNI coordinates, and smoothed using a 6-mm full-width-at-half-maximum Gaussian kernel.

A multiple regression analysis was performed using a general linear model to obtain statistical parametric maps that were calculated for the somatosensory stimulation. The fMRI signal time courses were high-pass filtered (128 s) and modeled as experimental-stimulus onset functions that were convolved with the canonical hemodynamic response function (low-pass filter). The individual results were projected onto their respective co-registered, high-resolution, T1-weighted, 3-D data sets. The anatomical localizations of the activated areas were analyzed with reference to the standard stereotaxic atlas and by visual inspection of the individual T1-weighted structural data. Individual maps were used to perform a random effect analysis using the standard summary statistic approach to obtain consistent group activation patterns. The resulting group statistical maps were thresholded according to the false discovery rate (FDR; *P* < 0.01).

### MEG Recordings

MEG was used to record magnetic fields with a 306-channel helmet-shaped neuromagnetometer (Vectorview, Elekta Neuromag Oy, Helsinki, Finland). The MEG data were sampled at 2 kHz and subsequently low-pass filtered at 1660 Hz and high-pass filtered at 0.1 Hz. A 3D digitizer (3SPACE FASTRAK, Polhemus Inc., Colchester, VT, USA) was used to identify the anatomical locations (i.e., preauricular points and nasion). Additional electrodes were positioned to capture the electrocardiographic (ECG) and electrooculographic (EOG) information.

### MEG Data Analysis

At the first preprocessing step, the recorded raw MEG data were filtered with Maxfilter version 2.0.21 (Elekta Neuromag Oy. Finland). The Maxfilter algorithm uses a signal space separation (SSS) method (Taulu and Simola, 2006). The further analysis of the MEG data was performed by using SPM12 software (Wellcome Department of Cognitive Neurology, London, UK^1^) and MATLAB (Mathworks, Natick, MA, USA). All recordings were visually inspected to detect the segments that were contaminated with noise. These segments were discarded from the subsequent analyses. We used the “Brainstorm” software (Tadel et al., 2011) for artifact correction. Heart and eye movement/blink contaminations were attenuated by designing the signal-space projections (SSP) from the selected segments of data related to each artifactual event (Nolte and Curio, 1999). Heartbeat and eye blink events were automatically detected in the ECG and EOG traces. The projectors were defined by applying principal component analysis (PCA) to these data segments. The data were orthogonally projected away from the principal component that best captured the artifacts’ sensor topographies. Further data processing was performed with SPM software. Data from all sensors were first high-pass filtered at 0.5 Hz and then low-pass filtered at 100 Hz. The data were then downsampled to 500 Hz and epoched from 50 ms before the stimulus onset to 300 ms after the stimulus onset. The pre-stimulus time window (−50 ms to 0 ms) was used for baseline corrections.

#### Model Specification

**Figure 3:**
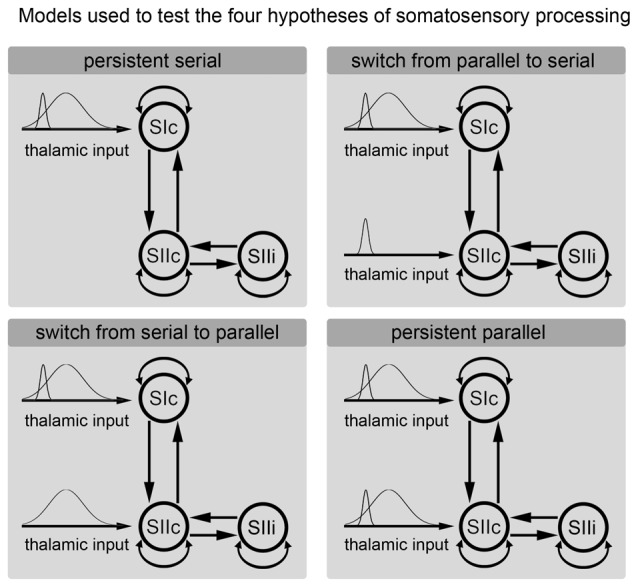
**Hypotheses regarding the processing of somatosensory information.** Each of the subfigures shows a model of the corresponding tested hypothesis. The extrinsic input functions of the model are shown to the left of each subfigure. The inputs correspond to stimulus functions encoding the tactile stimuli. These input functions are required by the implementation of the dynamic causal modeling (DCM) representing the thalamic input caused by the stimulus. Dependent on the model, these inputs are forwarded only to the primary somatosensory cortex (SI) or to both the SI and secondary somatosensory cortex (SII). The model of the cortical somatosensory network and its connections are illustrated to the right.

We used a DCM analysis to clarify whether somatosensory information is processed in parallel (directly entering the SII; Liang et al., 2011; Chung et al., 2014) or in series (entering only the SI and then further transmitted from the SI to the SII; Kalberlah et al., 2013; Khoshnejad et al., 2014). Specifically, we were interested in whether the processing route of somatosensory information changes over time. The cortical network consisted of three nodes (contralateral SI; contralateral SII; ipsilateral SII) that were activated during the fMRI experiment. The individual results of the fMRI analysis were used as prior locations for equivalent current dipoles in the modeling of the MEG data. The anatomical correctness of each location of a given region was further verified the using cytoarchitectonic probabilistic maps from the SPM Anatomy Toolbox (Eickhoff et al., 2005). We applied DCM for evoked responses using a network of standard event related potential (ERP) neural mass models (David et al., 2006). The evoked responses were spatially modeled by using a patch on the cortical surface (IMG in SPM software). The bidirectional connections were defined between the SIc and the SIIc and between the SIIc and the SIIi (Figure 3). Additionally, all nodes were modeled with self-connections.

Models that differed in terms of whether the stimulus information directly entered the SII node were compared. However, we were also interested in whether the processing route of the somatosensory information changed over time. Therefore, we divided the input signal from the thalamus into a phasic component that encoding the onset of a new stimulus and a sustained component that model the input during the duration of stimulus presentation (see Figure 3). We used Gaussian input functions as priors for the thalamic input with a mean of 30 ms/170 ms and a standard deviation of 16 ms/70 ms. This approach allowed us to test the following four models: (A) permanent serial information processing; (B) a switch from serial to parallel processing; (C) a switch from parallel to serial processing; and (D) permanent parallel processing of somatosensory information (Figure 3). For short time windows, the same analyses were performed with simplified models that modeled only the initial response (the start) of a stimulus and not the second input, which coded the sustained stimulus. The key directed connectivity parameters in this DCM corresponded to the strength of the coupling among nodes of the somatosensory system that are engaged by our (presumed) thalamic input. The models were specified and estimated using the DCM toolbox for SPM (SPM 12 release 6225). Usually, DCM assumes that the effective connectivity mediating evoked responses is fixed over the peri-stimulus time. Therefore, to address our hypothesis regarding changes in functional architecture (from serial to parallel or vice versa) we performed two complementary analyses that, reassuringly, yielded the same results. First, we modeled the entire peri-stimulus time window (up to 300 ms) using two stimulus-bound inputs representing early and sustained inputs. Importantly, these inputs could be deployed at high, low, or both levels of the somatosensory hierarchy, thereby allowing us to test whether one or both inputs were mediated by serial or parallel processing. This procedure allowed us to effectively dissociate serial and parallel processing in terms of early and late thalamic inputs. The second analysis employed a complementary approach in which we modeled the data over successive longer (or later) windows of stimulus time under two different models (serial and parallel) using the same thalamic input. For consistency, we applied the Bayesian model selection (BMS) approach to all windows used in the window selection approach. Our hypothesis here was that the predominant architecture would be reflected in the relative evidence for serial and parallel models and that this would change with the peri-stimulus duration.

#### Bayesian Model Selection and Comparison

The estimated DCMs were compared by using the model evidence. The model evidence is a measure of the probability of measuring the observed data given a particular model. The model evidence was compared at the group level in a random effect analysis (BMS). The details of this method are described elsewhere (Penny et al., 2010; Stephan et al., 2010). In short, a free energy approximation to the log evidence of each model is estimated in terms of the model fit and complexity (Friston et al., 2003). Based on the estimated model evidence, BMS calculates the probability that a given model is more likely than any other tested model to account for the data (Penny et al., 2010; Stephan et al., 2010). In the current study, BMS was separately performed for 18 data segments with lengths ranging from 40 ms to 260 ms that all began at 1 ms (Auksztulewicz et al., 2012). BMS was additionally performed with time windows of fixed lengths of 60 ms to further investigate the time course of a possible switch from parallel to serial information processing. We reported the exceedance probability (EP) for each tested model. The EP is a measure of the likelihood that one model is more likely (describes the data better) than any other model (Stephan et al., 2009; Penny et al., 2010). The EP sum to one over all models tested. For example, an EP of 90% means that we can be 90% confident that a specific model has a greater posterior probability than any other model. In the case of only two competing hypotheses, the EP is particularly intuitive as it describes the confidence that a model is more likely than the other one.

## Results

We applied DCM model comparison to clarify whether the processing of tactile information is performed in series or in parallel and whether there is a time-dependent change in the processing route. Figure 3 shows the four models that are compared in the current analysis. These models coding (A) sustained serial information processing; (B) a switch from parallel to serial processing; (C) a switch from serial to parallel processing; and (D) sustained parallel processing of somatosensory information (Figure 3).

### fMRI as a Spatial Localizer

**Figure 4:**
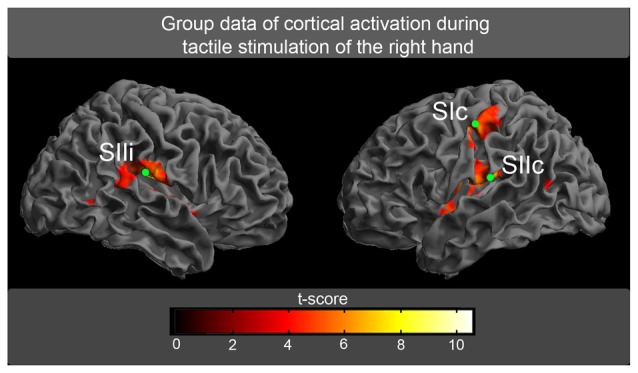
**The random-effect group analysis (*n* = 17) of BOLD responses.** Activations (*P* < 0.01, false discovery rate [FDR]-corrected) in response to tactile stimulation of fingers 1 + 3 of the right hand are shown superimposed on an inflated brain. The green circles correspond to the spatial locations of the maximum activation, which was further used for the magnetoencephalographic (MEG) analysis (SI, primary somatosensory cortex; SII, secondary somatosensory cortex, c, contralateral to tactile stimulation; i, ipsilateral to tactile stimulation).

The fMRI experiment was used to determine the spatial location of the areas of our model (SIc, SIIc, SIIi). The tactile stimulation of fingers 1 + 3 of the right hand evoked highly significant activations (*P* < 0.01, FDR-corrected) in the random-effect group analysis (Figure 4). These activations were located in the left SI and the bilateral SII (Table 1; Figure 4). The spatial locations of the activation maxima were further used in the DCM analysis of MEG data.

**Table 1:** **Cortical activation in response to tactile stimulation montreal neurological institute (MNI) coordinates of the activation maxima with corresponding *t*-values and standard deviation for right-sided tactile stimulation**.

Brain region	*x*	*y*	*z*	*t*-value
SIc	−57 ± 4.8	−19 ± 5.5	49 ± 6.2	9.8
SIIc	−48 ± 5.1	−28 ± 3.3	16 ± 3.5	10.5
SIIi	51 ± 7.2	−25 ± 5.2	19 ± 5.2	10.2

*SI, primary somatosensory cortex; SII, secondary somatosensory cortex; c, contralateral; i, ipsilateral*.

### DCM Model Comparison

**Figure 5:**
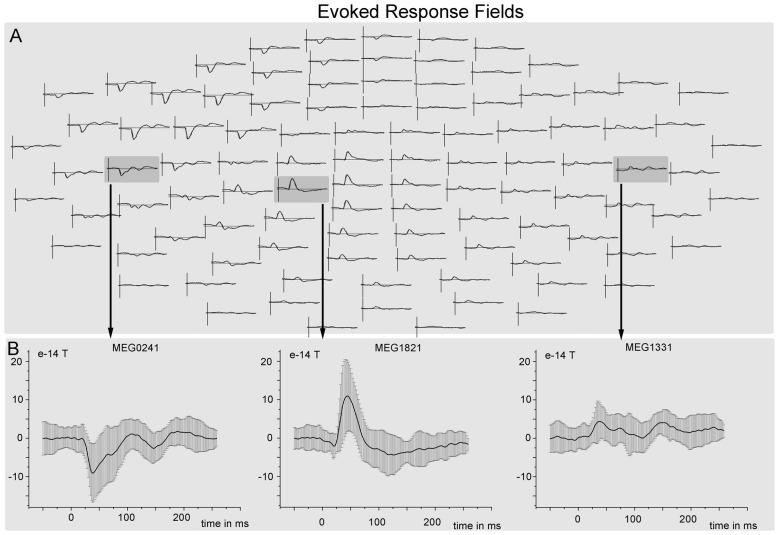
**Grand mean event related fields (ERFs). (A)** ERF responses (averaged over all trials and subjects) to tactile stimulation of the left hand overlaid on a whole scalp map of 102 MEG sensors. **(B)** ERF responses with standard deviation from three different sensors that are located nearest to the three sources (SIIc—sensor MEG0241, SIc—sensor MEG1821, SIIi—sensor MEG1331).

**Figure 6:**
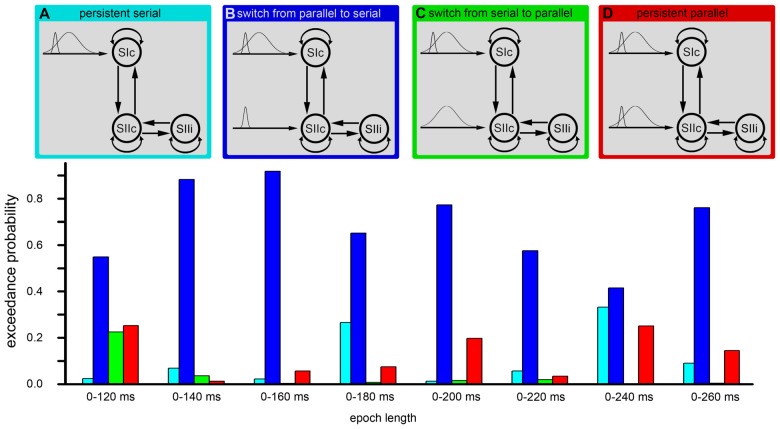
**DCM.** The figure shows the results of the Bayesian model selection (BMS). The four model structures are illustrated in the upper part of the image **(A–D)**. These four models were compared by the BMS. This model comparison was performed by estimating the exceedance probabilities (EP) for each model. The EP is a measure of the likelihood that one model is more likely than any other model. The model comprising a conversion from initially parallel to serial processing (second model from left/blue) was the most probable model in each time window.

The cortical network of the applied DCM model consisted of three nodes (contralateral SI, contralateral SII and ipsilateral SII) that were activated during the fMRI experiment. The grand average of the event related fields (ERFs) is shown in Figure 5. The use of DCM enabled us to test the four models shown in Figure 3. A random-effect Bayesian model comparison (*n* = 17) was applied to the four models over different time windows (Figure 6). The model representing a switch from parallel to serial processing (blue bars in Figure 6) clearly achieved the greatest EP value for all time intervals (Figure 6). A comparison of the strengths of the inputs revealed highly significant (*p* < 0.001) differences for all time intervals with stronger inputs in the SI than in the SII (not shown).

**Figure 7:**
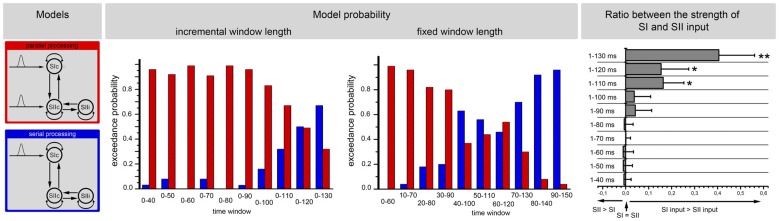
**The results of the BMS for the stimulus initiation.** The left side of the image illustrates the structures of the two models (i.e., parallel processing and serial processing). The middle illustrates the EP of these two models for 10 different time windows. The analysis was performed for incremental time windows (left side) and also for fixed time windows (right side of the middle part). The right side of the image illustrates the results of the posterior estimates for the most probable model (parallel input until 110 ms and serial input for the last two epochs) at the level of input strength. The input strengths in the SI and the SII are shown for the 10 different time windows. The *x*-axis represents the ratio between the strengths of the SI and SII inputs. Positive values represent stronger inputs in the SI than in the SII. The error bars represent the standard deviation. Significant differences between the SI and SII inputs are marked with “*” (*p* < 0.01) or “**” (*p* < 0.001).

The same analysis was additionally performed with simplified models that included only the initial response (the start) to the stimulus. Parallel and serial input models were compared (Figure 7). This analysis favored a parallel processing of information at the start of a new stimulus with a progressive change to a serial processing mode with increasing stimulus durations (Figure 7).

Additionally, we performed Bayesian model averaging to test for differences in the strengths of the inputs from the thalamus to the SI and from the thalamus to the SII. The Bayesian model averaging was performed for the winning model. Subject-specific input parameters were tested by a paired *t*-test. During the first 100 ms (parallel processing mode), we did not find significant differences in the strength between the SI and the SII, while for longer time windows, we found a stronger thalamic input to the SI compared to the thalamic input to the SII (Figure 6). However, these results did not indicate that the switch between parallel and serial processing occurs at approximately 100–130 ms because the analyses involve the complete time-course from the start of the stimulus. To further investigate the timing of the processing switch, we added a further analysis with a constant window length of the analyzed signal. Here, we used a window length of 60 ms (Figure 6). Note that we can model small windows of responses, several 100 ms after stimulus onset, because we have an explicit forward or generative (dynamic causal) model of how neuronal sources response to inputs. While the first three fixed time windows (until 30–90 ms) indicated a parallel processing mode, the results of the next four time windows were not conclusive but indicated a shift from parallel to serial processing. Afterwards, a serial processing mode dominated (Figure 6). These results did not allow for determining the exact timing of the switch in the processing mode, but indicated that a switch occurred between 60 ms and 120 ms.

**Figure 8:**
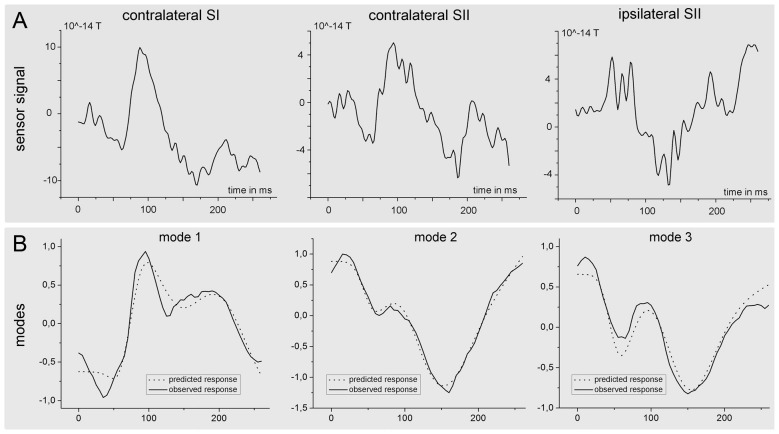
**Time course of the source activity and sensor readings of a single subject.** The upper row **(A)** of the figure shows the measured signal at the sensor level for one single subject. The signal was estimated as the mean (preprocessed) signal of the four sensor channels nearest to the source. The lower part **(B)** of the figure shows predicted (dotted) and observed (solid) responses in measurement space for the first three spatial modes, which were obtained after projection of the sensor data onto seven spatial modes.

The results of the Bayesian model averaging were further used to extract the time-course of the source activity of the three nodes (SIc, SIIc and SIIi) at the individual level. Figure 8 shows the preprocessed data at the sensor level of one subject together with the predicted (dotted) and observed (solid) responses in measurement space for the first three spatial modes of the same subject.

## Discussion

In the present study, we applied DCM in combination with BMC (Penny et al., 2010) to investigate the architecture of the processing route of somatosensory information with a specific focus on the time dependence of this architecture. Our results strongly support an initial parallel processing pathway in which somatosensory information directly enters the SII that is followed by a processing stream, which is dominated by serial processing.

The results did not disagree with the classical thinking that the main flow of somatosensory information projects from the thalamus to the SI and then further to the SII. In the SI, somatosensory information is preprocessed, i.e., the intensity, duration, location, size, shape and type of a somatosensory stimulus are encoded, from the contralateral half of the body (Schnitzler and Ploner, 2000; Zhang et al., 2007; Klingner et al., 2010). This preprocessed information is then transmitted to the SII. However, the dominance of this processing pathway does not exclude the coexistence of direct projections from the thalamus to the SII. Our results suggest that such direct input to the SII and the corresponding parallel processing of information is the dominant processing mode during the first 100 ms following a somatosensory stimulus. A parallel pathway would also explain the observation of early responses in the SII (at 20–30 ms) following somatosensory stimulation (Karhu and Tesche, 1999). A parallel processing mode was further supported by a study in marmosets in which reduced but preserved SII responsiveness was observed following inactivation of the SI (Rowe et al., 1996). SII responsiveness was abolished in ~10%, reduced in 65% and unaffected in 25% of SII neurons (Rowe et al., 1996). Particularly, the reduced responsiveness in the majority of neurons suggests that the neurons not only receive direct input from the thalamus but also input from the SI. Combining these results with our findings regarding the change in the processing mode from an early parallel processing mode to a serial processing mode led us to the hypothesis that there are neurons in the SII that process both direct inputs from the thalamus while later receiving and processing inputs from the SI. As an alternative explanation, it is also possible that those SII neurons that receive direct thalamic input undergo fast habituation, while the thalamic input remains stable. In both cases, the increasing input from the SI to the SII leads to a shift in the processing mode to primarily serial information processing, and direct thalamic input to the SII does not significantly contribute to explaining the observed data as time progresses. Consequently, an alteration in the processing stream would be expected at the time at which preprocessed information from the SI enters the SII. However, this exact time point is unknown. Studies that have investigated response timing in the SII have come to divergent conclusions, reporting response times of 20–30 ms (Karhu and Tesche, 1999; Inui et al., 2004) and up to 90 ms (Hagiwara et al., 2010). However, with respect to the current results, the responses in the SII could also have been generated by direct input from the thalamus. Therefore, it remains unclear whether the early potentials in the SII measured by these studies are caused by input from the SI or the thalamus. Correspondingly, the analysis of the early potentials in the SII has not conclusively answered the question of when SI activity enters the SII. It can be assumed that information is transmitted from the SI to the SII once the preprocessing of this information in the SI is completed. However, it is known that the preprocessing of somatosensory information in the SI is performed at different stages in the different subareas of the SI (Rojas-Hortelano et al., 2014; Sathian, 2016). It can therefore be assumed that information that needs only minor preprocessing (i.e., only preprocessing in BA3b) are transferred and arrive in the SII prior to information that undergoes preprocessing requiring more time. Therefore, the amount of transmitted information should progressively increase over a certain time interval. We suggest that this time window is represented by the switch of the dominant processing mode from parallel to serial. However, because all the models incorporated data from the initial parallel processing, the switch in information processing should occur before the switch in the models (i.e., prior to 120 ms). The additional analysis with fixed time windows confirmed this upper time limit while suggesting a start of the switch in the processing mode between 60 ms and 90 ms.

Further arguments for the necessity of a switch from parallel to serial information flow can be derived from studies of tactile working memory. It was previously demonstrated that the memory trace of a tactile stimulus is held not only in the prefrontal cortex and the posterior parietal cortex (Romo et al., 1999; Kaas et al., 2013) but also in the SI (Zhou and Fuster, 1996; Harris et al., 2001; Pasternak and Greenlee, 2005; Kaas et al., 2013; Wang et al., 2013). The presence of memory cells in the SI agrees well with the theory that memory is stored in the same cortical system that participates in the processing of these sensory information (Squire and Zola-Morgan, 1991). The SI therefore has dual functions: to encode sensory stimuli and to store this information for short amounts of time (Zhou and Fuster, 1996; Pasternak and Greenlee, 2005; Wang et al., 2013). It was further shown that the SI retains tactile memory traces for at least as long as the tactile stimulus was applied (Harris et al., 2002). The retrieval, comparison and adaptation of memory traces in the SI with information from the SII additionally increase the amount of information transferred between both areas and increase the importance of the serial pathway. However, the storage and retrieval of tactile memory traces does not occur earlier than the specific feature is encoded by the SI. Therefore, this mechanism does not add to the serial information flow shortly after the stimulus onset. We hypothesize that the time between stimulus onset and stimulus encoding in the SI corresponds to the time period that is dominated by a parallel processing pathway.

The purpose of a switch in the processing mode remains to be elucidated. Here, we attempt to discuss this topic by appealing the theory of predictive coding. This theory interprets evoked cortical responses as transient expressions of prediction errors. The recognition of stimuli is thought to be a process of minimizing the prediction errors at all stages of cortical hierarchy (Mumford, 1992; Rao and Ballard, 1999; Friston, 2005). Higher order cortical areas are assumed to estimate and transmit predictions for lower-level neural activities by feedback connections. The forward connections carry the residual error between the predictions and the actual lower-level activities. An abrupt change in our somatosensory environment (stimulus) causes a strong prediction error. The minimization of this error can be performed by altering the neuronal states of different levels of the somatosensory processing hierarchy, which could be achieved by simply using the forward and backward connections between the cortical areas. However, direct input to the SII allows for more precise estimation of the prediction error at an earlier time point. This prediction error influences the information processing in the entire hierarchical network that is mediated by forward, lateral and backward connections and ultimately results in faster error minimization, i.e., the recognition of the cause of a stimulus. This indicates that a direct input to the SII is reasonable only if fast information processing of thalamic information in the SII is of importance. It is conceivable that this applies to the quick identification of a stimulus (e.g., to identify the dangerousness of a stimulus). Whether a stimulus is dangerous not only depends on the stimulus intensity but is also strongly context-dependent and requires the context of a hierarchical organized network that includes higher-order cognitive resources (e.g., to identify the salience of a stimulus). Whether the stimulus is salient or not depends also on the currently available cognitive resources. This context sensitivity is mediated by precision weighting in predictive coding, which we now consider in more detail.

In the absence of a significant prediction error (steady state stimulus), we suggest that direct thalamic input is further used to screen for discrepancies relative to the information delivered by the SI. However, in the absence of an information discrepancy, we suggest that further processing mainly relies on the preprocessed SI information and not on the direct thalamic input to the SII. In the absence of new influences in an unaltered sensory environment, the serial processing mode becomes dominant. Pursuing the predictive coding explanation, one simple and potentially important explanation for our findings rests upon the attentional selection of somatosensory prediction errors in the SI by the SII. In brief, predictive coding rests upon ascending prediction errors that are weighted by their precision (or reliability). This boosting of precise prediction errors has been discussed in terms of attentional gain, particularly in the visual system (Brown and Friston, 2012, 2013). Critically, precision must be predicted in a top-down fashion to mediate attentional selection. This means that the SII needs to know which prediction errors to select prior to their subsequent (hierarchical) processing. In this sense, our results fit comfortably with the idea that thalamic inputs to the SII enable descending connections from the SII to the SI to set the appropriate precision or post-synaptic gain and contextualize subsequent (serial) processing based on a reciprocal exchange of ascending prediction errors and descending predictions.

The current observation of a change in the processing mode may explain recent conflicting results from fMRI-DCM analyses (Liang et al., 2011; Kalberlah et al., 2013; Chung et al., 2014; Khoshnejad et al., 2014). The parallel processing of information should be found to be the prominent mode based on analyses that focus mainly on the perception of a change in the somatosensory environment, e.g., analyses based on data acquired shortly after stimulus onset or perhaps based on the use of only stimuli. However, the translation of such a fast processing change into hemodynamic responses is insufficiently understood and is therefore difficult to interpret. Our results, in conjunction with the available fMRI-DCM studies, strongly suggest that both an initial parallel transfer of information and a subsequent serial transfer of information dominate the processing of somatosensory information. It is tempting to speculate regarding whether direct thalamic input to higher level brain areas is an exclusive feature of the SII or rather a more general attribute of brain architecture. Specifically, it is unknown whether other areas that are involved in the processing of somatosensory information (e.g., the insula) receive direct thalamic input. If parallel input decreases the time required to make an inference about the cause of a sensory stimulus, similar architectures would be expected at least in other sensory systems.

In addition to thalamic input, it would be interesting to determine whether similar time-dependent changes in processing modes might also be present in other functional systems in which one area is in need of information from another area only at a certain point in time to minimize the prediction error. Such connections might be shut down later during resting states or may be completely lost in data with poor temporal resolution. Moreover, information processing is well known to be hierarchically organized, and the dynamic alterations of information streams by top-down and bottom-up modulations are feasible (Friston et al., 2003; Garrido et al., 2007). Although our understanding of functional connections has greatly improved over the past decade, there is a lack of knowledge regarding the temporal dynamics of the transfer of information between brain areas that are known to be connected. This deficit highlights the importance of the use of methods that provide high temporal resolution.

## Conclusion

The current study investigated the processing route of tactile information in the somatosensory cortex. Our results suggest a time-dependent change in the processing stream of information corresponding to ongoing somatosensory information from an initial parallel processing to a subsequent serial processing.

## Author Contributions

CMK, SB, RH and OWW have written and edited the manuscript together. CMK, SB and RH were involved in data generation and analysis. CMK and OWW were involved in the design and concept of the current study.

## Conflict of Interest Statement

The authors declare that the research was conducted in the absence of any commercial or financial relationships that could be construed as a potential conflict of interest.

## Footnotes

^1^http://www.fil.ion.ucl.ac.uk/spm
